# Atlas of Iberian water beetles (ESACIB database)

**DOI:** 10.3897/zookeys.520.6048

**Published:** 2015-09-16

**Authors:** David Sánchez-Fernández, Andrés Millán, Pedro Abellán, Félix Picazo, José A. Carbonell, Ignacio Ribera

**Affiliations:** 1 Institut de Biologia Evolutiva (CSIC-Universitat Pompeu Fabra), Passeig Maritim de la Barceloneta 37-49, 08003, Barcelona, Spain; 2 Departamento de Ecología e Hidrología, Universidad de Murcia, Campus de Espinardo, 30100, Murcia, Spain; 3 Department of Biology, Queens College, City University of New York. 65-30, Kissena Blvd Flushing, NY 11367, USA

**Keywords:** Aquatic, Coleoptera, freshwater, Iberian peninsula, occurrence, Portugal, Spain

## Abstract

The ESACIB (‘EScarabajos ACuáticos IBéricos’) database is provided, including all available distributional data of Iberian and Balearic water beetles from the literature up to 2013, as well as from museum and private collections, PhD theses, and other unpublished sources. The database contains 62,015 records with associated geographic data (10×10 km UTM squares) for 488 species and subspecies of water beetles, 120 of them endemic to the Iberian Peninsula and eight to the Balearic Islands. This database was used for the elaboration of the “Atlas de los Coleópteros Acuáticos de España Peninsular”. In this dataset data of 15 additional species has been added: 11 that occur in the Balearic Islands or mainland Portugal but not in peninsular Spain and an other four with mainly terrestrial habits within the genus *Helophorus* (for taxonomic coherence). The complete dataset is provided in Darwin Core Archive format.

(‘EScarabajos ACuáticos IBéricos’)

## General description

**Purpose**: The purpose of this paper is to provide all the available distributional information on water beetles from the Iberian Peninsula and Balearic Islands. These data were compiled in the ESACIB (‘EScarabajos ACuáticos IBéricos’) database and published in the “Atlas de los Coleópteros Acuáticos de España Peninsular” (Millán et al. 2014). However, there are some mismatches between the species presented in Millán et al. (2014) and those presented in this dataset (see the section “additional information” for details). Water beetles have high species richness in the Mediterranean region, inhabiting virtually every kind of fresh and brackish water habitat, from the smallest ponds to lagoons and wetlands, and from streams to irrigation ditches, large rivers, and reservoirs (e.g. Ribera et al. 1998, Ribera 2000, Millán et al. 2002). In comparison to other groups of freshwater invertebrates in the Iberian peninsula and the Balearic islands, water beetles are well known in their systematics and biogeography (Ribera et al. 1998, Ribera 2000, Millán et al, 2006). In this context, the ESACIB database was developed to provide all the available distributional information on water beetles from this region. This database represents the most complete information available for a major group of freshwater invertebrates in the study area.

**Additional information**: The species included in this dataset but not considered in Millán et al. (2014) are:

i) species endemic to the Balearic Islands (*Deronectes brannanii* (Schaufuss, 1869); *Hydroporus lluci* Fery, 1999; Hydraena (Hydraena) balearica Orchymont, 1930; *Graptodytes kuchtai* (Breit, 1908); *Limnebius minoricensis* Jäch, Valladares & García-Avilés, 1996; Ochthebius (Ochthebius) javieri Jäch, 2000; Ochthebius (Ochthebius) pedroi Jäch, 2000; *Oulimnius echinatus* Berthélemy, 1979);

ii) species endemic to mainland Portugal (*Rhithrodytes agnus agnus* Foster, 1992; *Rhithrodytes agnus argaensis* Bilton & Fery, 1996; Hydraena (Hydraena) malagricola Jäch & Díaz, 2012; Hydraena (Hydraena) optica Jäch & Díaz, 2012; Hydraena (Hydraena) zezerensis Díaz Pazos & Bilton, 1994);

iii) species present in mainland Portugal (*Porhydrus vicinus* (Aubé, 1838)) or the Balearic Islands (*Ochthebius lobicollis* Rey, 1885) but not in the Iberian mainland;

iv) for taxonomic coherence four species of Helophorus (subgenus Empleurus) are included in this dataset that were not treated in Millan et al. 2014 due to their mostly terrestrial habits (Helophorus (Empleurus) hispanicus (Sharp, 1915); Helophorus (Empleurus) porculus (Bedel, 1881); Helophorus (Empleurus) rufipes (Bosc, 1791); Helophorus (Empleurus) schmidti A. Villa & G.B. Villa, 1838).

It should be noted that some Iberian species such as *Macronychus quadrituberculatus* P.W.J. Müller, 1806, Haliplus (Haliplus) sibiricus Motschulsky, 1860, *Berosus bispina* Reyche & Saulcy 1856, *Helophorus cincticollis* Guillebeau, 1893 and *Hydraena assimilis* Rey, 1803 are not included in this dataset due to the lack of geographical precision of their records in the study area. In addition, part of the data presented here are also included in the “Inventario Español de Especies Terrestres (MAGRAMA)”.

## Project details

**Project title**: Atlas de los coleópteros acuáticos de España peninsular

**Personnel**: Andrés Millán (IP), David Sánchez-Fernández (co-IP), Pedro Abellán, Félix Picazo, José A. Carbonell, Jorge M. Lobo, Ignacio Ribera.

**Funding**: This project was funded by the Spanish Ministry of Agriculture, Alimentation and Environment (MAGRAMA). Some data have been obtained with the support of additional projects from the Spanish Government, in particular 023/2007, CGL2007-61665 and CGL2013-48950.

**Study area descriptions/descriptors**: The Iberian peninsula and Balearic islands are two closely bio-geographically related areas which extend more than 585,644 km^2^. The territory includes a variety of biomes, relief, climates, and soil types, where altitude ranges from sea level to 3483 m.a.s.l. in the Sierra Nevada (SE Iberia). These areas are of great biogeographic interest, being regarded as one of the richest European regions in terms of species diversity (Domínguez-Lozano et al. 1996, Médail and Quézel 1999, Reyjol et al. 2007). Insects in general, and beetles in particular, make up the highest percentage of the biodiversity of this area. Close to 98% of the total Iberian fauna are invertebrates, and roughly 81% are insects (Ramos et al. 2001). The Iberian peninsula has a wide range of habitat types, including some aquatic environments very rare in Europe (Millán et al. 2011). Some of these freshwater ecosystems are subjected to strong human influence, and are in consequence under risk of suffering high rates of biodiversity loss (Allan and Flecker 1993, Saunders et al. 2002).

**Design description**: The database compiles all available taxonomic and distributional data of the families of strictly aquatic Coleoptera from the literature as well as from museum and private collections, PhD theses, and other unpublished sources. The bibliographic references providing more records are mainly papers compiling distributional data for several families (Fery and Fresneda 2007), PhD thesis (Valladares 1988, Garrido 1990, Millán 1991), and some regional catalogues (Rico 1996, Ribera et al. 1996, Millán et al. 2002, Sánchez-Fernández et al. 2003). It is note worthy that the single most important source of records of this dataset was the IBE collection (Institut de Biologia Evolutiva, CSIC-UPF, Barcelona), with more than 7000 records.

## Data published through

GBIF: http://www.gbif.es/ipt/resource?r=esacib

## Taxonomic coverage

**Figure 1.**
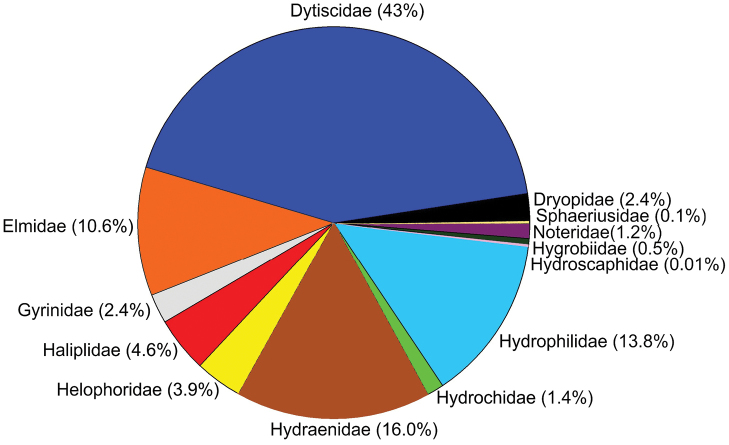
Taxonomic distribution of the dataset (percentage of species per family).

**General taxonomic coverage**: We focus here exclusively on the “strictly aquatic beetles”, i.e., those that spend most of their life submerged, at least in its adult stage, in any type of aquatic ecosystem (Jäch and Balke 2008). More concretely, we focus on 13 families of water beetles belonging to three suborders (Fig. 1).

## Taxonomic ranks

Kingdom: Animalia

Phylum: Arthropoda

Class: Insecta

Order: Coleoptera

Suborder: Myxophaga, Adephaga, Polyphaga

Family: Hydroscaphidae, Sphaeriusidae, Dytiscidae, Gyrinidae, Haliplidae, Hygrobiidae, Noteridae, Dryopidae, Elmidae, Helophoridae, Hydraenidae, Hydrochidae, Hydrophilidae

## Spatial coverage

**Figure 2.**
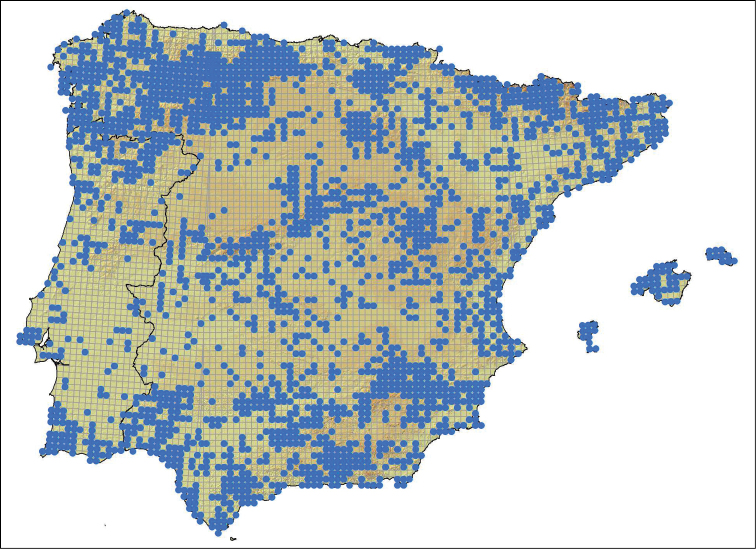
Geographic distribution of the records.

**General spatial coverage**: The study area is the Iberian peninsula (Spain and Portugal mainland) and Balearic islands, located in the southwest of Europe (Fig. 2).

**Coordinates**: 35°23'60"N and 43°58'48"N Latitude; 10°2'24"W and 4°48'36"E Longitude.

**Figure 3.**
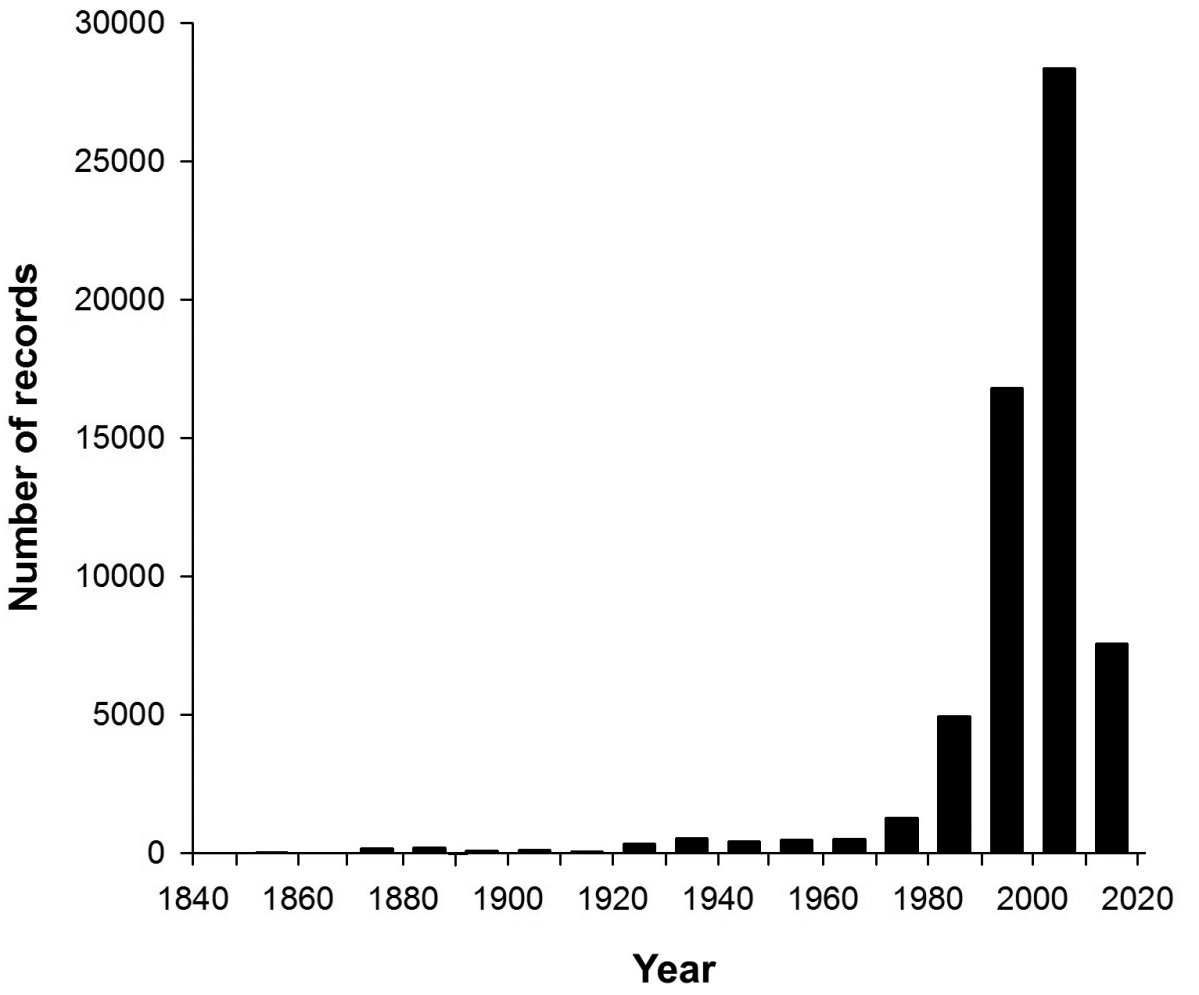
Temporal distribution of the records.

**Living time period**: 1840–2013 (Fig. 3).

## Methods

**Method step description**:

1. Primary sources of the data

1a. Sampling

1b. Bibliographic compilation

1c. Public and private collections

2. Georeferenciation at 10×10 km grid cells

Records were assigned to 10×10 km grid cells based on the Universal Transverse Mercator (UTM) projection and the Military Grid Reference System (MGRS) from the spatial coordinates provided in the original sources. In those cases in which records did not attach spatial coordinates, the locations of the records were identified in Google Earth, translated to UTM coordinates and assigned to 10×10 km grid cells. Those records that could not be unambiguously georeferenced were discarded.

3. Introduction in the database.

4. Elaboration of distributional maps.

5. Checking for doubtful records.

6. Modifications of records (taxonomy or coordinates).

7. Elaboration of new distributional maps.

**Study extent description**: The Iberian peninsula and Balearic islands. The frequency of sampling has been irregular, as data were obtained from bibliographic sources, field sampling, and the revision of private collections.

**Sampling description**: For the unpublished data, in most cases at each sampling site beetles were collected from a representation of all mesohabitat types with a kick-net of 500 µm mesh, following in most cases a multihabitat protocol (Jáimez-Cuéllar et al. 2002). Each kick-sample was examined in the field and successive samples were taken until no new morpho-types were found. The kick-sample contents were pooled into a unique site-sample, preserved in 70% or 96% ethanol and identified to species level in the laboratory.

**Quality control description**: Distributional maps for each species were generated that were checked by all members of the project and some external reviewers. Doubtful records were double-checked (identifications, geographical coordinates, etc.). In the case of doubtful data from published sources, the original papers were reviewed again, and in some cases (whenever possible) additional information was requested from the authors on the doubtful records.

## Datasets

### Dataset description

**Object name**: Darwin Core Archive Atlas of Iberian water beetles (ESACIB database)

**Character encoding**: UTF-8

**Format name**: Darwin Core Archive format

**Format version**: 1.0

**Distribution**: http://www.gbif.es/ipt/archive.do?r=esacib

**Publication date of data**: 2015-04-29

**Language**: English

**Licences of use**: To the extent possible under law, the publisher has waived all rights to these data and has dedicated them to the <a href=”http://creativecommons.org/publicdomain/zero/1.0/legalcode”>Public Domain (CC0 1.0)</a>. Users may copy, modify, distribute and use the work, including for commercial purposes, without restriction.

**Metadata language**: English

**Date of metadata creation**: 2015-01-26

**Hierarchy level**: Dataset
